# Apparent Power Law Scaling of Variable Range Hopping Conduction in Carbonized Polymer Nanofibers

**DOI:** 10.1038/srep37783

**Published:** 2016-11-25

**Authors:** Kyung Ho Kim, Samuel Lara-Avila, Hojin Kang, Hans He, Johnas Eklӧf, Sung Ju Hong, Min Park, Kasper Moth-Poulsen, Satoshi Matsushita, Kazuo Akagi, Sergey Kubatkin, Yung Woo Park

**Affiliations:** 1 Department of Physics and Astronomy, Seoul National University, Seoul, 08826, Korea; 2 Department of Microtechnology and Nanoscience, Chalmers University of Technology, SE-412 96 Gothenburg, Sweden; 3 Department of Chemistry and Chemical Engineering, Chalmers University of Technology, SE-412 96 Gothenburg, Sweden; 4 Department of Polymer Chemistry, Kyoto University, Katsura, Kyoto 615-8510, Japan

## Abstract

We induce dramatic changes in the structure of conducting polymer nanofibers by carbonization at 800 °C and compare charge transport properties between carbonized and pristine nanofibers. Despite the profound structural differences, both types of systems display power law dependence of current with voltage and temperature, and all measurements can be scaled into a single universal curve. We analyze our experimental data in the framework of variable range hopping and argue that this mechanism can explain transport properties of pristine polymer nanofibers as well.

Conductive polymers are an attractive route to cost effective and versatile nanoelectronics. Chemical synthesis of these materials offers possibility to chemically bottom-up engineer the electronic function of polymers at will, and yet produce them in large scale. As of now, several proof of concept devices based on conductive polymers have been shown that span a great range of applications including flexible field-effect transistors^1^, actuators^2^, sensors^3^, and nano-optoelectronic devices^4^. Insight in fundamental electron transport properties in these materials is of utmost importance for further practical developments.

While electron transport properties of macroscopic conductive polymer films have been widely studied and reported in literature^5^, the low dimensional -1D- counterpart is less explored. Currently there is no consensus on the dominant transport mechanism in 1D or quasi 1D polymer conductors. In principle, polymer nanofibers are simple quasi 1D systems composed of weakly coupled 1D chains, making them attractive test beds to study electron transport in low dimensions. In practice, ubiquitous disorder in real samples complicates the analysis of electron transport in the context of well-established models. Nonetheless, refinements in chemical synthesis of polymers have contributed to greater reproducibility of experimental data on transport across experiments. For instance, a variety of polymer nanofibers including polyacetylene (PA), polypyrrole (PPy), polyaniline (PANI), and polythiophene (PT) have been shown to display power law dependence of conductance in temperature, *G*(*T*) ∝ *T*^*α*^ and current-voltage characteristic, *I*(*V*) ∝ *V*^*β*^ in a wide range of *T* and *V* parameters^6^,^7^. Electrical transport through polymer nanofibers including such power law dependence can be explained, within some limited range of *T* and *V*, in the framework of variable range hopping (VRH)^8^, fluctuation induced tunneling (FIT)^9^, electric field induced tunneling^10^,^11^,^12^, manifestation of Luttinger liquid (LL) and environmental Coulomb blockade (ECB)^6^,^7^. Still, none of the aforementioned mechanism could describe precisely the fine details of data in the entire range of parameters.

Seemingly, unification of electron transport in conducting polymers, be it films or quasi-1D systems, arises in an expression formula frequently used to fit the macroscopic current in conductive polymers:


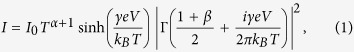


where *I*_0_, *α*, *β*, *γ* are the fitting parameters, *k*_*B*_ is the Boltzmann constant, *e* is the electron charge, and Γ(*x*) is the gamma function^6^,^7^,^13^,^14^,^15^,^16^. The expression for macroscopic current described by Eq. 1 has its origin in the microscopic description of electron transfer rates for dissipative tunneling in Caldeira-Leggett models^17^,^18^,^19^. In a nutshell, these entail an asymmetric double well potential, in which the tunneling rates from the metastable well slightly higher in energy is driven by the coupling to a full phonon bath. The fitting parameters in the tunneling rate expression have physical meanings; *α* and *β* are related to the dissipation coupling strength between charge and the phonon bath, and *I*_0_ ∝ Δ^2^/*ω*^*α*+2^, where Δ is the tunneling frequency (electron coupling between initial and final states) and *ω* is the characteristic frequency in the well^15^,^17^,^18^. The functional form of Eq. 1 has been observed to describe variety of 1D or quasi-1D systems^6^,^7^,^20^,^21^,^22^,^23^. Remarkably, the scaling has also been observed beyond 1D systems in conducting polymer films such as poly(2,5-bis(3-tetradecylthiophen-2-yl) thieno[3,2-b]thiophene) (PBTTT)^13^,^14^,^15^ and poly(3,4-ethylenedioxythiophene)-poly(styrenesulfonate) (PEDOT:PSS)^14^,^16^, and theories such as polaron hopping in semiconducting polymers^15^, ECB^16^, and VRH^24^,^25^,^26^ have been developed to explain these findings. More generally, Eq. 1 is also useful in the phenomenological description of macroscopic quantum tunneling, such as Josephson currents in superconductors, and the tunneling of the magnetic flux within the ring of superconducting quantum interference devices (SQUIDs)^27^,^28^. Thus, Eq. 1 appears as a very versatile expression that encompasses the physics of a variety of systems and transport mechanism. Interpretation of experimental results of transport measurements in the context of Eq. 1 is carried out together with a priori knowledge of the system under study^6^,^7^,^13^,^14^,^15^,^16^,^20^,^21^,^22^,^23^,^24^,^25^,^26^. Depending on the context, one can assign a meaning to the fitting parameters. For instance, in the case of VRH, *γ*^*−*1^ is related to *L*/*ξ,* where *ξ* is the localization length and *L* is the sample length, and *α* is related to number of hops^24^. In the context of electron tunneling into LL, *γ*^*−*1^ is the number of tunneling barriers with the constraints that *β *= *α* + 1 and *γ*^−1^* *= 1 in single tunneling^6^,^7^,^13^,^20^,^21^,^22^,^29^; for polaron hopping mediated by nuclear tunneling in semiconducting polymers, *γ*^−1^ represents the number of hopping events needed for the charge carriers to traverse the distance between electrodes and *β* = *α* + 1^15^. These models have all in common *α*, the exponent of the temperature dependence of conductivity, *G*(*T*) ∝ *T*^*α*^ and *β*, the exponent of the voltage dependence of current, *I*(*V*) ∝ *V*^*β*^.

In this work we probe electrical transport properties of nanofibers of the conducting polymers, trans-PA and PANI, and study the effect of carbonization in the context of Eq. 1. The quasi one-dimensional structure of as-produced polymer nanofibers is dramatically modified by pyrolysis at *T* = 800 °C, transforming the original polymer structure into quasi-amorphous carbon networks, yet retaining its nanofibrila morphology^30^,^31^,^32^,^33^. Despite the profound modifications to the polymer structure, we find remarkably similar transport properties in the nanofibers before and after carbonization by means of current-voltage (*I*-*V*) measurements in the range 1.5 K < *T* < 300 K: highly non-linear *I*-*V* characteristics, power law dependence of *I*(*V*) ∝ *V*^*β*^ at high bias voltages (*eV*/*k*_*B*_*T* ≫ 1) and *G*(*T*) ∝ *T*^*α*^ at low voltages (*eV*/*k*_*B*_*T* ≪ 1), and universal scaling according to Eq. 1. We argue that the unexpected similarity of transport between pristine and carbonized polymers emphasize the importance of carrier localization in polymer nanofibers, and that the transport mechanism of polymer fibers for the past reports in literature is hopping like. Qualitatively, the crossover between the transport regimes, *G*(*T*) ∝ *T*^*α*^ and *I*(*V*) ∝ *V*^*β*^, occurs at voltages where the carrier’s electrical energy for a single hop *eV*_*hop*_ is comparable to the carrier’s thermal energy *k*_*B*_*T*. When *eV*/*k*_*B*_*T* ≪ 1, the current is Ohmic with power law dependence on temperature, and when *eV*/*k*_*B*_*T* ≫ 1, the current is superlinear in voltage weakly dependent on temperature.

## Results

**Figure 1:**
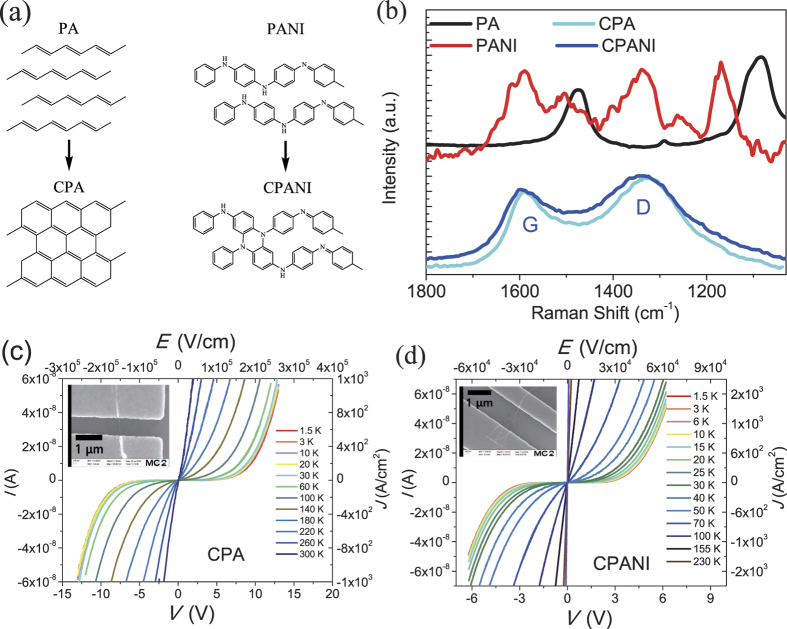
Structural differences between pristine and carbonized polymers and *I*-*V* characteristics of CPA and CPANI nanofibers. (**a**) Possible cross-linking of PA (adapted from ref. 30) and PANI (adapted from refs 33 and 34) after carbonization at 800 °C. (**b**) Raman spectra of PA and PANI nanofibers show characteristic peaks of trans-PA at 1475 and 1085 cm^−1^ and complex peaks of HCl doped PANI at 1620, 1590, 1505, 1405, 1340, 1260, and 1170 cm^−1^. After carbonization, broad graphite G and disordered D bands were observed in both CPA and CPANI nanofibers. Data for PA and PANI are normalized with respect to the intensity of D band and shifted for clarity. (**c,d**) *I*-*V* (*E*-*J*) characteristics of CPA (**c**) and CPANI (**d**) nanofibers. Inset figures are scanning electron microscope (SEM) images of CPA (**c**) and CPANI (**d**) nanofibers with Ti/Au top contact electrodes.

Figure (1a) shows the possible cross-linked structure of both carbonized polyacetylene (CPA) and carbonized polyaniline (CPANI) upon pyrolysis at 800 °C as suggested in literatures^30^,^33^,^34^. According to the literature, cross-linking of molecular chains upon pyrolysis leads to the partially graphite-like^30^ and the partially phenazine-like^33^,^34^ structure for CPA and CPANI, respectively. Figure (1b) shows the Raman spectra (*λ* = 638 nm) measured on bundles of pristine (iodine doped PA and hydrogen chloride (HCl) doped PANI) and carbonized polymer nanofibers on Si/SiO_2_ substrates. Before carbonization, we observed characteristic Raman peaks of trans-PA at 1475 and 1085 cm^−1^ [Fig. (1b)]^35^ which are assigned to C = C stretching vibration and mixed vibration of C-C stretching and C-H in-plane bending, respectively^31^,^35^,^36^. For HCl doped PANI nanofibers, complex characteristic peaks at 1620, 1590, 1505, 1405, 1340, 1260, and 1170 cm^−1^ were observed [Fig. (1b)]^34^. After carbonization, both nanofibers show broad bands which have local maximum at 1320 (1340) cm^−1^ and 1590 (1600) cm^−1^ for CPA (CPANI) which are assigned to disordered (D) and graphitic (G) bands of graphite, corresponds to the breathing mode of aromatic rings and the stretching motion of sp^2^ carbon atoms, respectively^30^,^31^,^32^,^33^,^37^. The broad linewidths (FWHM > 100 cm^−1^) of the G band and the relative intensities of D and G bands *I*_*D*_/*I*_*G*_ = 1.2 in both carbonized fibers indicate that the sp^2^ cluster size is smaller than 1 nm^38^,^39^. The small sp^2^ crystallite size in carbonized polymers, and the evidence by previous XRD^30^,^31^,^32^, TEM^40^ and spectroscopic studies^30^,^31^,^32^,^33^,^34^ on PA^30^,^31^,^40^ and PANI^32^,^33^,^34^ carbonized under the same conditions, support that carbonization of polymers yields partially cross-linked polymer chains forming quasi-amorphous carbon networks [Fig. (1a)]. The carbonized polymers are not crystallized yet as the graphitization occurs at 2600 °C^30^,^31^,^32^, which means that carbonized polymers are quasi-amorphous networks of hexagonal carbon bonds.

Figure 1(c,d) shows that both CPA [Fig. 1(c)] and CPANI [Fig. 1(d)] fibers display qualitatively the same transport properties in the temperature and voltage ranges of this study. The *I*-*V* characteristics are highly non-linear at low temperatures and there are transport gaps with threshold voltages of ~2.5 V (5 × 10^4^ V/cm) for CPA and 1 V (1 × 10^4^ V/cm) for CPANI nanofibers at 1.5 K. For temperatures below *T* < 10 K, the current voltage characteristic is nearly temperature independent in both CPA and CPANI nanofibers. The devices of Fig. 1(c,d) consist of individual carbonized nanofibers with typical diameters in the range 15–80 nm and 15–55 nm, respectively for CPA and CPANI. The typical length of nanofibers was 10 *μ*m, which facilitates defining several electrical contacts by standard lithography methods. The carbonized fibers of Fig. 1(c,d) have been contacted with Ti/Au top electrodes; no difference is observed if Pd or Ni/Au are used instead as confirmed by comparison of two-probe and four-probe measurements. The room-temperature, two-probe conductivity of 15 devices with CPANI (0.03–2 S/cm) was found to be somewhat larger than that measured in 21 devices with CPA (0.01–0.1 S/cm). If we compare the conductivity of carbonized fibers with that of individual pristine fibers, we find that CPA displays an order of magnitude lower conductivity than iodine-doped PA nanofibers (0.01–100 S/cm), but that the conductivity of CPANI is comparable with that of HCl doped PANI nanofibers (0.1–1 S/cm)^7^.

**Figure 2:**
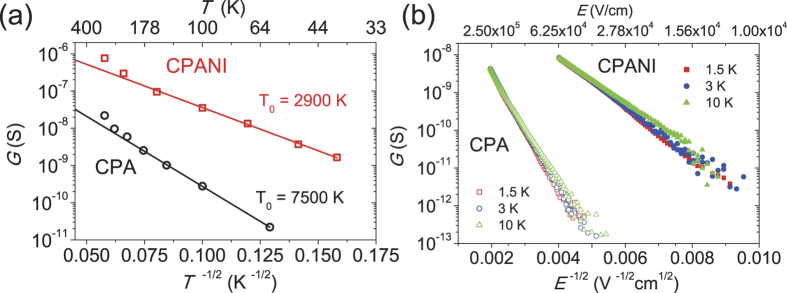
ES-VRH behaviors of CPA and CPANI nanofibers. (**a**) The temperature dependence of the Ohmic conductance at low bias voltages follows the ES-VRH, *G*(*T*) ∝ exp[−(*T*_0_/*T*)]^1/2^ at *T* < 180 K and deviates from the ES-VRH at *T* > 180 K. (**b**) The bias electric field dependence of the non-Ohmic conductance at low temperatures (*T* < 10 K) and high voltages (*V* > *~* 1 V) shows the characteristic of the ES-VRH, *G*(*E*) ∝ exp[−(*E*_0_/*E*)]^1/2^.

Analysis of the non-linear *I*-*V* in the two limiting transport regimes of the low (*eV*/*k*_*B*_*T* ≪ 1) and the high bias (*eV*/*k*_*B*_*T* ≫ 1) voltages reveals that the electron transport in these system is of conventional VRH^41^,^42^. Figure 2 shows that the conductance, *G* = *I*/*V* in both the low bias Ohmic regime and the high bias non-linear regime follows the characteristic *T* and *V* dependence of Efros-Shklovskii VRH (ES-VRH)^43^, respectively. At low bias and *T* < 180 K, the *I*-*V* of both CPA and CPANI is Ohmic and well described by *G*(*T*) ∝ *exp*[−(*T*_0_/*T*)]^1/2^ [Fig. (2a)], where *T*_0_ is the characteristic temperature with *T*_0_ = 7500 K for CPA and *T*_0_ = 2900 K for CPANI fibers. This temperature dependence is attributed to the ES-VRH, in which a Coulomb gap is formed due to the long-range Coulomb interaction of the electron-hole pair created during charge carrier hopping^43^. At *T* > 180 K, deviation from the ES-VRH law is prominent in both fibers. We ascribe the deviation at high temperatures to the contribution of short-range Coulomb interactions which is excluded in the ES-VRH and dominant at high energies. Screening of long-range interactions at high temperatures and crossover to a distinct temperature relation due to the contribution of the short-range interaction to the gap have been raised recently in strongly correlated materials and perovskite-type compounds^44^. Moreover, at high bias voltages (*V* > ~ 1 V) and low temperatures (*T* < 20 K), the conductance of both CPA and CPANI is almost temperature independent and follows, *G*(*E*) ∝ *exp*[−(*E*_0_/*E*)]^1/2^, where *E* is the electric field and *E*_0_ = *k*_*B*_*T*_0_/2*eξ* is the characteristic electric field with *E*_0_ = 1.2 × 10^7^ V/cm (*E*_0_ = 2.5 × 10^6^ V/cm) for CPA (CPANI) nanofiber [Fig. (2b)]. This inverse square root voltage dependence is expected for the ES-VRH in the limit *eEξ* ≫ *k*_*B*_*T* and *ξ* = 0.3 nm (0.5 nm) for CPA (CPANI) was obtained by combining *T*_0_ and *E*_0_, which is consistent with the analysis of Raman spectroscopy^45^,^46^,^47^.

**Figure 3:**
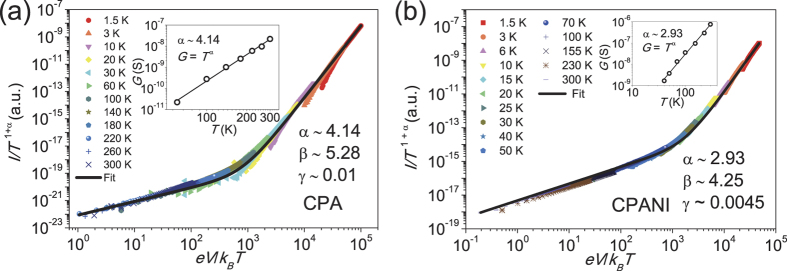
Universal scaling of CPA and CPANI nanofibers. *I*/*T*^1+ *α*^ vs *eV*/*k*_*B*_*T* plot of both CPA (**a**) and CPANI (**b**) nanofibers. Inset shows power law dependence of Ohmic conductance, *G*(*T*) ∝ *T*^*α*^ in both CPA (**a**) and CPANI (**b**) nanofibers. Fit to Eq. 1 (black curves) gives fitting parameters *I*_0_, *β*, and *γ*.

With the ES-VRH behavior in the limited range of *T* and *V*, Eq. 1 gives the opportunity to describe the data in the entire range of parameters of our study. Remarkably, it is hard to distinguish between the stretched exponential temperature dependence, *G*(*T*) ∝ *exp*[−(*T*_0_/*T*)]^1/2^ and the power law temperature dependence, *G*(*T*) ∝ *T*^*α*^ in low bias Ohmic regime due to the limited range of temperature. If we plot the Ohmic conductance in log-log scale, the Ohmic conductance mimics power law behavior *G*(*T*) ∝ *T*^*α*^ with *α* = 4.14 for CPA and *α* = 2.93 for CPANI nanofibers [Inset of Fig. 3(a,b)]. Similar is the non-Ohmic *I*-*V* at low temperatures as shown in Fig. 3. This means that the apparent power law *G*(*T*) ∝ *T*^*α*^ [Inset of Fig. 3(a,b)] and *I*(*V*) ∝ *V*^*β*^ (Fig. 3) in certain parameter ranges might arise from the ES-VRH. Using the power law exponent *α*, we plotted all *I*-*V* curves for all temperatures as *I*/*T*^1+ *α*^ vs *eV*/*k*_*B*_*T* according to Eq. 1 and found that all *I*-*V* curves collapse into a single curve for both CPA and CPANI [Fig. 3(a,b)]. The fitting parameters using Eq. 1 are *γ*^−1^ = 100 for CPA and *γ*^−1^ = 205 for CPANI nanofibers. Remarkably, these values are comparable (same order of magnitude) with number of tunneling barriers (*γ*^−1^) obtained in pristine PA and PANI polymer nanofibers^7^ (summarized in Table 1).

## Discussion

Having in mind the considerable disorder and the ES-VRH behavior of carbonized polymer fibers, we interpreted the apparent power law scaling in carbonized polymer fibers within the framework of VRH^24^,^25^,^26^. We considered the 1D-VRH model developed by Rodin and Fogler^24^, the electric field compensated VRH model proposed by Li *et al*.^25^, and the implementation of effective temperature concept in VRH proposed by Abdalla *et al*.^26^ as possibilities. Since carbonized polymers have undergone cross-linking of polymer chains, the explanation of Li *et al*. and that of Abdalla *et al*. which do not necessarily include one-dimensional systems are more appropriate to explain our data. Li *et al*. have explained the physical origin of the power law scaling by invoking electric field compensation for thermal activation in VRH in wide range of *T* and *V* parameters. Abdalla *et al*. have shown that the scaling of Eq. 1 is phenomenologically equivalent to the scaling function of the effective temperature, 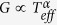. Physically, the effective temperature concept combines the effects of the high electric field and the lattice temperature, which is reminiscent of the model of Li *et al*. Importantly, Abdalla *et al*. have reproduced the same scaling by numerical simulation incorporating Coulomb interactions in the Miller-Abrahams expression of hopping rates. In the same fashion, we propose that the power law scaling in carbonized polymer fibers is originated from the ES-VRH between local sp^2^ carbon sites in the networks of quasi-amorphous carbon bonds.

**Figure 4:**
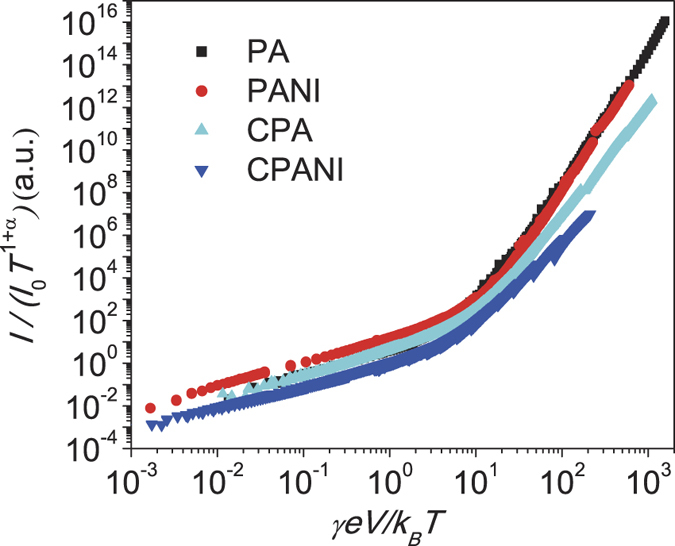
Comparison of universal scaling between pristine and carbonized polymer nanofibers. Normalized power law scaling *I*/*I*_0_*T*^*α*+1^ vs *γeV*/*k*_*B*_*T* of both pristine and carbonized polymer nanofibers shows similar transport in these systems. Crossovers of slopes in all scaled curves occur when *γeV/k*_*B*_*T* ~ 1.

Less trivial to explain is the fact that the similarities of transport properties go beyond carbonized fibers and extend to pristine PA and PANI fibers, despite the fact that the molecular structure of polymers is dramatically modified after carbonization. Figure 4 shows a direct comparison of transport properties for both carbonized PA and PANI nanofibers (Fig. 4) and those of pristine polymers shown in ref. 7. When transport measurements are plotted as *I*/*I*_0_*T*^*α*+1^ vs *γeV*/*k*_*B*_*T*, all curves from different polymer nanofibers show the same scaling behaviors before and after carbonization. We note that crossover of slopes in all scaled curves occur when *γeV/k*_*B*_*T* ~ 1, similar to those of polymer films in ref. 15.

The similarity of transport between pristine and carbonized polymer nanofibers suggests the need to revise the transport mechanism in pristine polymer nanofibers. The universal scaling, *I*/*T*^*α*+1^ vs *eV*/*k*_*B*_*T* observed in pristine polymer fibers^6^,^7^ was proposed as an evidence of 1D transport in polymer fibers, with the assumption that the building block for such nanofiber structures consist of inherently-1D polymer chains. In previous reports, authors propose that conductivity in pristine polymer nanofibers arise from of intra-chain transport (i.e. tunneling along 1D polymer chains), where each chain is a collection of Luttinger-liquid-like islands separated by intramolecular tunneling barriers^6^,^7^. This conclusion was drawn solely from the observed power law variations of *G*(*T*) and *I*(*V*) and universal scaling, *I*/*T*^*α*+1^ vs *eV*/*k*_*B*_*T*. However, in these systems is not obvious why in principle one should expect LL behavior, given that the pristine polymers are neither perfectly clean nor truly 1D, not to mention that the conditions for the fit to Eq. 1 in the context of LL, *β* = *α* + 1 and *γ*^−1^ = 1, are not met^6^,^7^. Polymer fibers differ from single molecule oligomers^48^ in that one fiber consists of large number of molecular chains stacked at best in polycrystalline structures. Moreover, and in comparison with this work, the same universal scaling (Eq. 1) as well as the power law variations of *G*(*T*) and *I*(*V*) are observed in carbonized polymer fibers, in which the initially 1D polymer chains have undergone partial cross-linking upon pyrolysis forming quasi-amorphous networks of hexagonal carbon bonds [Fig. 1(a)]. The networking of polymer chains is expected to lessen the intra-chain transport (i.e. tunneling along 1D polymer chains), originally considered as transport mechanism in polymer nanofibers. Therefore, instead of tunneling of delocalized carriers along the polymer chains, proposed for pristine PA fibers^6^,^7^, transport in pristine polymers might as well occur due to hopping between localized states.

In the ES-VRH interpretation of Eq. 1 in both pristine and carbonized polymer fibers, *α* and *β* are related to the dissipation coupling strength of an electron to the phonon bath; *γ*^−1^ is related to the number of hops needed for the charge carriers to traverse the distance between electrodes. Therefore, fitting parameters of Eq. 1 provide intuition for the charge transport of the system. Temperature and voltage dependence of conductance become faster as the coupling strength *α* and *β* increase because tunneling from metastable well slightly higher in energy is driven by the coupling to the phonon bath. Large number of *γ*^−1^ in carbonized fibers indicates large number of hops due to strong disorder. The disordered structure presumably arises from defects during dehydrogenation and cross-linking processes. Moreover, the aromatic rings formed by carbonization are not crystallized yet^30^,^31^,^32^. Therefore, order of magnitude comparable values of *γ*^−1^ in pristine polymers suggests that the structural disorder in stacking of molecular chains in pristine polymer fibers is significant.

In the interpretations of our measurements in carbonized polymer fibers, one can argue that the threshold voltage at low temperatures [Fig. 1(c,d)] might also result from Coulomb blockade (CB) effects. The theory of CB transport in metallic dot arrays by Middleton and Wingreen predicts a current dependence, *I* ∝ (*V* − *V*_*t*_)^*ζ*^ with *ζ* *~* 1 in 1D, and *ζ* *~* 2 or 5/3 in 2D^49^. However, the exponent *ζ* is usually larger than the theoretical value due to topological inhomogeneity^50^ as we observed 2.4 < *ζ* < 4.1 (See Fig. S16). We considered the self-capacitance *C*_0_ = 4*πεε*_0_*r* of a crystalline island of radius *r* and the mutual capacitance *C*_*i*_ = 2*πεε*_0_ln[(*r* + *d*)/*d*], where 2*d* is the spacing between crystalline islands^51^,^52^. The radius *r* = 0.5 nm is used from localization lengths and Raman spectroscopy analysis and 2*d* + 2*r* = *γL* gives *d* = 2 nm for both CPA and CPANI. Then, the charging energy 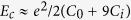 is about 350 K, with dielectric constant ε ~ 20^51^,^52^. This means that the threshold voltage should exist even at room temperature, which contradicts to our data. The threshold voltage exists below *T* < 100 K (*T* < 30 K) for CPA (CPANI) nanofibers. Therefore, instead of CB, we ascribe the threshold voltage to the crossover from activation-less hopping to phonon emission hopping. Also the contact resistance is small compared to the sample resistance (See Fig. S17) and the *I*-*V* is symmetric in two probe geometry. Therefore, the contribution from the Schottky barrier to the non-linear transport is negligible compared to the sample resistance.

## Conclusion

In conclusion, we have analyzed the transport data of the carbonized polymers in the framework of the ES-VRH and have compared charge transport in pristine and carbonized polymer nanofibers in the context of Eq. 1. Despite the different structure, confirmed by Raman spectroscopy, both types of materials give apparent power law dependence of current with voltage and temperature and scaling of all measurements into a single universal curve. We have interpreted the power law scaling in carbonized polymer nanofibers as the manifestation of the ES-VRH in wide range of *T* and *V* parameters, which we suggest as the main transport mechanism of pristine polymer nanofibers as well.

## Methods

### Polymer nanofiber synthesis

Polymer nanofibers have been produced according to standard protocols. The aligned PA film was synthesized from acetylene gas of six-nine grade using a nematic liquid crystal as a solvent for the Ziegler-Natta catalyst, Ti(O-n-Bu)_4_/AlEt_3_. The concentration of [Ti] was 50 mmol/l and the mole ratio of the co-catalyst, [Al]/[Ti], was 4. The nematic liquid crystal was the equimolar mixture of two kinds of phenylcyclohexyl derivatives, *para*-(trans-4-n-propylcyclohexyl) ethoxybenzene (PCH302) and *para*-(trans-4-n-propylcyclohexyl)butoxybenzene (PCH304). The nematic liquid crystal containing the catalyst was aligned at 10 °C through gravity flow to make an aligned reaction field on the glass wall of a Schlenk flask. The polymerization was carried out at 10 °C on the reaction field by introducing the acetylene gas for about 30–53 min into the flask. The initial pressure of the acetylene gas was 509–511 Torr. The aligned PA film synthesized was washed with distilled toluene, methanol solution containing 1 N hydrochloric acid, and distilled toluene in turn at room temperature under flowing argon gas, and then was dried in vacuum on a Teflon sheet^53^. In the case of PANI, HCl doped PANI nanofibers were synthesized by rapidly mixing aqueous acidic solution of ammonium peroxydisulfate with aqueous acidic solution of aniline and catalytic amount of *p*-phenylenediamine as a promoter for fiber growth. 3.2 mmol of aniline monomers was added in 10 mL of 1 N hydrochloric acid and 5 mg of *p*-phenylenediamine was dissolved in small amount of methanol. The two solutions were mixed and the resultant solution was added to the solution of 0.8 mmol of Ammonium peroxydisulfate in 10 mL of 1 N hydrochloric acid. The resulting solution was violently shaken for 10 seconds and left for 1 day^54^.

### Carbonization of polymer nanofibers

The aligned PA film was dispersed in *N*,*N*-Dimethylformamide (DMF) with ultra-sonication without surfactant more easily than helically entangled PA film due to the entanglement-free morphology^55^. The dispersed PA nanofibers were drop casted on 6 × 6 mm^2^ Si/SiO_2_ (300 nm) substrates and doped by gaseous iodine for one hour. Iodine doping of PA films increase carbonization yield in the subsequent pyrolysis step, preventing thermal decomposition by promoting cross-linking of PA chains upon hydrogen iodide gas removal^30^,^31^. PANI nanofibers were dispersed on Si/SiO_2_ (300 nm) by drop casting. Pyrolysis of fibers for both drop casted PA and PANI nanofibers took place in a tube furnace at 800 °C for 1 hour under nitrogen flow with 1 °C/min of both heating and cooling ramp rate.

### Raman and electrical measurement

Structural characterization was carried out by Raman spectroscopy, performed on bundles of pristine polymer fibers (iodine doped PA and HCl doped PANI) and on carbonized polymer fibers drop casted on Si/SiO_2_ substrates using a Horiba scientific Raman spectrometer equipped with a spotsize ~1 *μ*m (*λ* = 638 nm). For electrical measurement, Ti/Au (5/95 nm), Pd (50 nm), and Ni/Au (50/50 nm) electrodes were defined on top of the nanofibers by conventional e-beam lithography using poly(methylmethacrylate) (PMMA) as a positive resist. The electrical integrity of the Si/SiO_2_ dielectric during the high temperature step was verified at room temperature with measurements in four/two-probe geometries with a Keithley Semiconductor Characterization System 4200 (SCS 4200). Temperature dependence of the *I*-*V* characteristic was measured in two-probe geometry with a Keithley 6517 electrometer using an Oxford Instruments Maglab system equipped with a Variable Temperature Insert (VTI) for measurements at temperatures between 1.5 K and 360 K.

## Additional Information

**How to cite this article**: Kim, K. H. *et al*. Apparent Power Law Scaling of Variable Range Hopping Conduction in Carbonized Polymer Nanofibers. *Sci. Rep.* **6**, 37783; doi: 10.1038/srep37783 (2016).

**Publisher's note:** Springer Nature remains neutral with regard to jurisdictional claims in published maps and institutional affiliations.

## Supplementary Material

Supplementary Contents

## Figures and Tables

**Table 1:** Fitting parameters for pristine and carbonized polymer nanofibers.

	*α*	*β*	*I*_0_(*A*/*K*^1+*α*^)	*γ*^−1^
PA^7^	5.47	6.88	4.9 × 10^−23^	40
CPA	4.14	5.28	3.0 × 10^−21^	100
PANI^7^	5.34	6.28	8.3 × 10^−23^	303
CPANI	2.93	4.25	1.0 × 10^−15^	205
