# Author Correction: Apparent Power Law Scaling of Variable Range Hopping Conduction in Carbonized Polymer Nanofibers

**DOI:** 10.1038/s41598-020-62904-0

**Published:** 2020-04-10

**Authors:** Kyung Ho Kim, Samuel Lara-Avila, Hojin Kang, Hans He, Johnas Eklӧf, Sung Ju Hong, Min Park, Kasper Moth-Poulsen, Satoshi Matsushita, Kazuo Akagi, Sergey Kubatkin, Yung Woo Park

**Affiliations:** 1 Department of Physics and Astronomy, Seoul National University, Seoul, 08826 Korea; 2 Department of Microtechnology and Nanoscience, Chalmers University of Technology, SE-412 96 Gothenburg, Sweden; 3 Department of Chemistry and Chemical Engineering, Chalmers University of Technology, SE-412 96 Gothenburg, Sweden; 4 Department of Polymer Chemistry, Kyoto University, Katsura, Kyoto 615-8510 Japan

Correction to: *Scientific Reports* 10.1038/srep37783, published online 25 November 2016

This Article contains a typographical error in Equation 1.

$$I=\,{I}_{0}{T}^{\propto +1}\,\sinh \left(\frac{\gamma eV}{{k}_{B}T}\right){|\Gamma \left(\frac{1+{\rm{\beta }}}{2}+\frac{i\gamma eV}{2\pi {k}_{B}T}\right)|}^{2}$$

should read:

$$I=\,{I}_{0}{T}^{\propto +1}\,\sinh \left(\frac{\gamma eV}{2{k}_{B}T}\right){|\Gamma \left(\frac{1+{\rm{\beta }}}{2}+\frac{i\gamma eV}{2\pi {k}_{B}T}\right)|}^{2}$$

