# Supplementary material for: Apparent Power Law Scaling of Variable Range Hopping Conduction in Carbonized Polymer Nanofibers
Source: Sci Rep. 2016 Nov 25;6:37783. doi: 10.1038/srep37783 (PMC5122886; doi:10.1038/srep37783)
Supplement: Supplementary Contents [file srep37783-s1.pdf]

Supplementary Contents of

Apparent Power Law Scaling of Variable Range Hopping Conduction in Carbonized Polymer Nanofibers

Kyung Ho Kim,[1] Samuel Lara-Avila,[2] Hojin Kang,[1] Hans He,[2] Johnas Eklöf,[3] Sung Ju Hong,[1] Min Park,[1] Kasper Moth-Poulsen,[3] Satoshi Matsushita,[4] Kazuo Akagi,[4] Sergey Kubatkin,[2] and Yung Woo Park[1,*]

[1]*Department of Physics and Astronomy, Seoul National University, Seoul, 08826, Korea*

[2]*Department of Microtechnology and Nanoscience, Chalmers University of Technology, SE-412 96 Gothenburg, Sweden*

[3]*Department of Chemistry and Chemical Engineering, Chalmers University of Technology, SE-412 96 Gothenburg, Sweden*

[4]*Department of Polymer Chemistry, Kyoto University, Katsura, Kyoto 615-8510, Japan*

*Corresponding authors: ywpark@phya.snu.ac.kr

1. Measurements of the CPA-1 nanofiber

Figure S1 is the AFM topography of the CPA-1 nanofiber with Ti/Au (5/95 nm) top contact electrodes. Diameter of the CPA-1 nanofiber is ~70 nm and the distance between electrodes is 500 nm determined by AFM topography. Room temperature resistance and conductivity of the CPA-1 nanofiber was 40 MΩ and 0.03 S/cm.

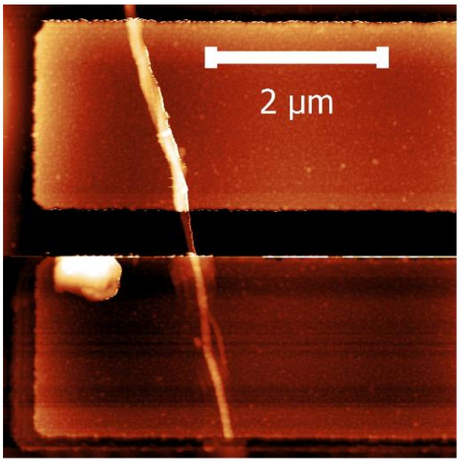

Figure. S1. AFM topography of the CPA-1 nanofiber (denoted by CPA in paper) and with Ti/Au (5/95 nm) top contact electrodes. Diameter of the nanofiber is ~70 nm and the distance between electrodes is 500 nm.

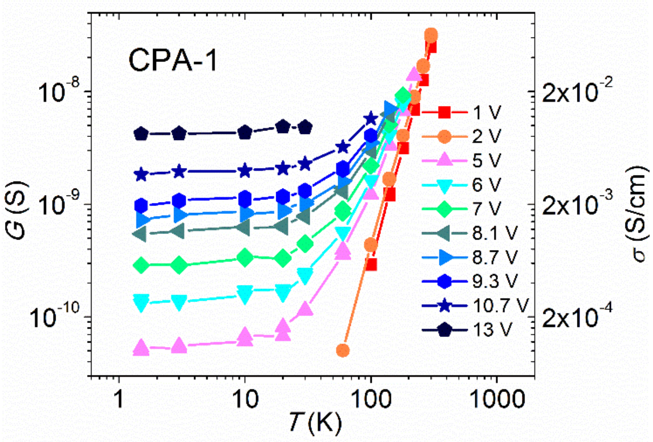

Figure. S2. Temperature dependence of conductance at various fixed voltages of the CPA-1 nanofiber is presented in log-log scale. At 1 and 2 V, conductance strongly depends on temperature and follows power law. As voltage increases and temperature lowers, temperature dependence tends to decrease, which supports the hopping transport where activation-less hopping occurs at low temperature and electric field compensates for activation energy at high voltages.

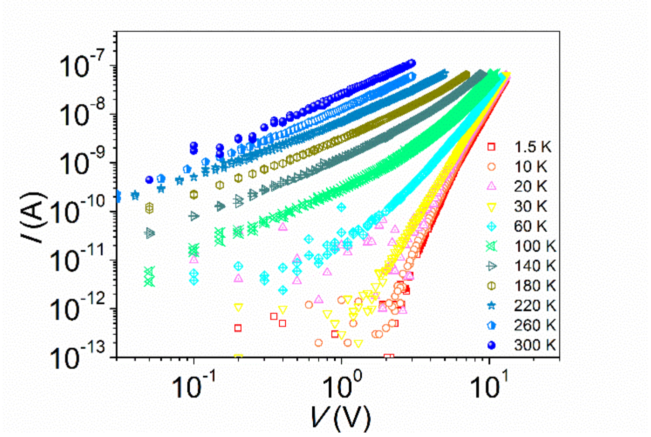

Figure. S3. *I*-*V* of the CPA-1 nanofiber in log-log scale follows apparent power law behaviors. The power law at low temperatures is apparent after threshold voltages at T < 30 K.

2. Measurements of CPA-2 and CPA-3 nanofibers

Figure S4 is AFM picture of the CPA-2 (A-B) and the CPA-3 nanofibers (C-D). Electrodes (A, B, C, D) of Ti/Au (5/95 nm) were contacted on top of the nanofibers and distance between electrodes are 500 nm, 1.84 μm for A-B and C-D, respectively. Diameter of the CPA-2 (CPA-3) nanofiber is 60 nm (25 nm) and a cross-junction between the CPA-2 and the CPA-3 nanofibers is formed between electrode B-C and B-D.

Figure S(5a) shows the temperature dependence of I-V characteristics. It shows similar behaviors as that of the CPA-1 nanofiber such as non-linearity, transport gap and weak temperature dependence at low temperatures. Other similar behaviors observed in the CPA-1 nanofiber are also observed in the CPA-2 nanofiber; Power law behavior of conductance at low bias and its deviation at high bias voltages [Figure S(5b)], quasi power law behavior of I-V [Figure S(5c)] and scaling behavior of I-V curves [Figure S(5d)]. Room temperatureresistance and conductivity of the CPA-2 nanofiber was 30 MΩ and 0.04 S/cm, similar with those of the CPA-1 nanofiber (40 MΩ, 0.03 S/cm).

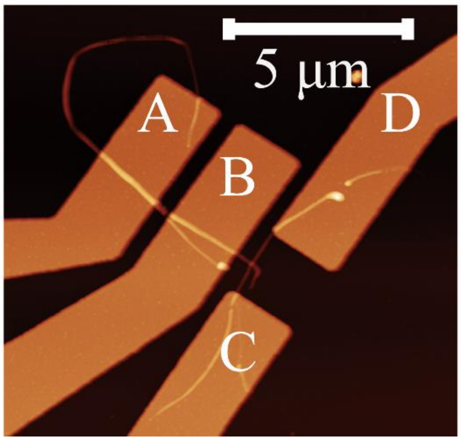

Figure. S4. AFM picture of the CPA-2 nanofiber (A-B) and CPA-3 nanofiber (C-D) with Ti/Au (5/95 nm) top contact

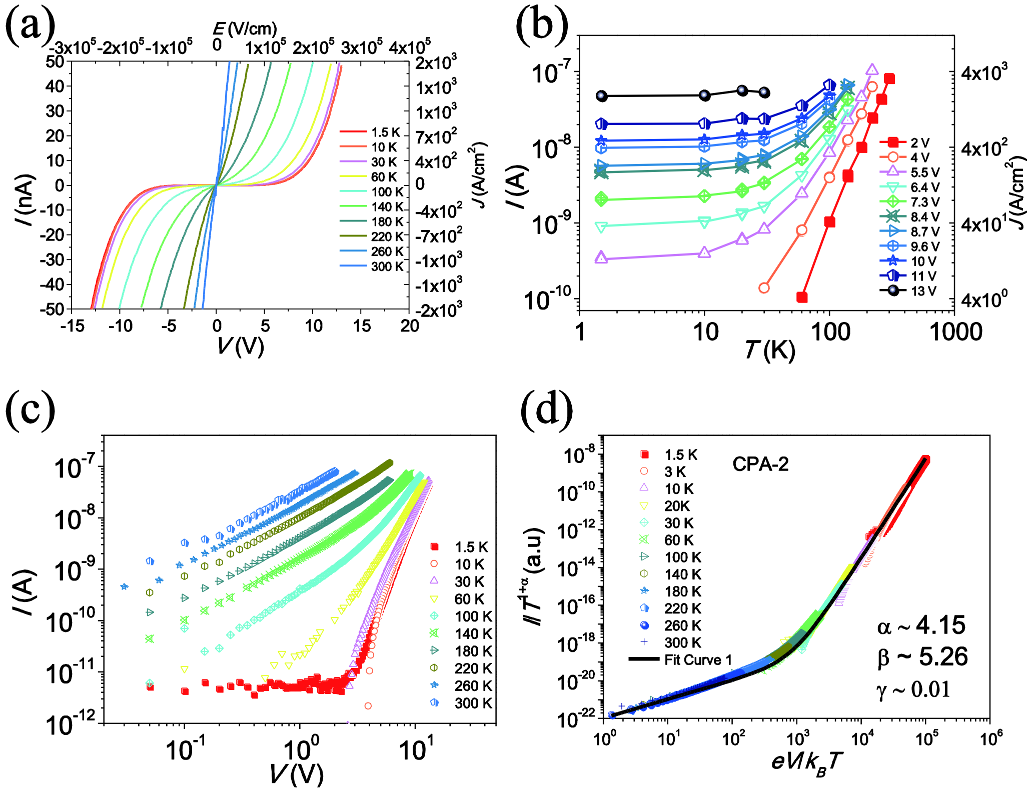

Figure. S5. (a) Temperature dependence of *I-V* characteristics for the CPA-2 nanofiber in linear scale. (b) Temperature dependence of current at various source-drain applied voltages. As voltage increases temperature dependence weakens and deviates from power law dependence at low voltages. (c) Temperature dependence of *I-V* characteristics plotted in double logarithmic scale shows quasi power law behavior. (d) *I-V* curves from different temperatures collapsed in a single universal curve. α is the exponent of the temperature dependence of conductivity, $G(T) \propto T^{\alpha}$ and β is obtained from the fitting.

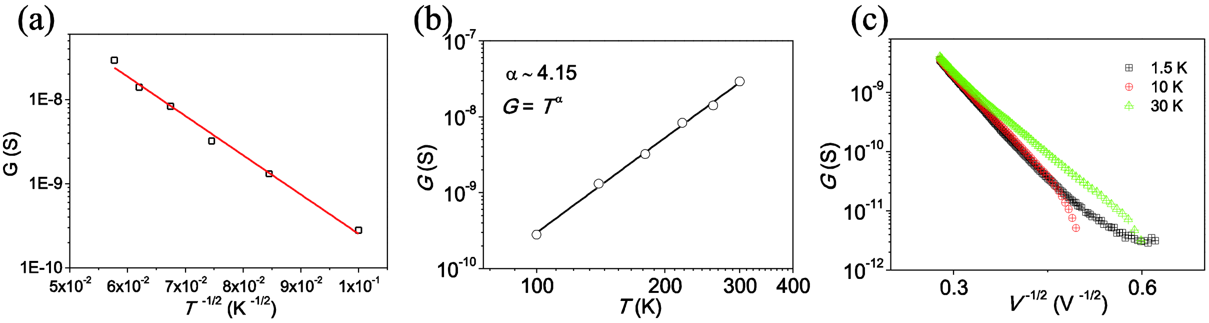

Figure. S6. The temperature dependence of the Ohmic conductance of the CPA-2 nanofiber at low bias voltages are plotted as, $G(T) \propto \exp\left[-(T_0/T)^{1/2}\right]$ (a) and power law (b). The characteristic temperature was calculated as T_0 = 11500 K. (c) The bias voltage dependence of the non-Ohmic conductance at low temperatures (T < 30 K) and high voltages (V > ~1 V) shows the characteristic of ES-VRH, $G(V) \propto \exp\left[-(T_0/V)^{1/2}\right]$ in the CPA-2 nanofiber.

Figure S(7a) shows temperature dependence of I-V characteristics for CPA-3 nanofiber. For CPA-3 nanofiber, the inter-electrode length was relatively long and room temperature resistance was relatively high (240 MΩ, 0.16 S/cm). However, it shows similar behaviors as

those of the CPA-1 and the CPA-2 nanofibers such as non-linearity, transport gap and weak temperature dependence at low temperatures. Moreover other behaviors observed in CPA-1, CPA-2 nanofibers such as power law behavior of conductance at low bias and weaker temperature dependence at high voltages [Figure S(7b)], quasi power law behavior of I-V [Figure S(7c)] and scaling behavior of I-V curves [Figure S(7d)] are also observed in the CPA-3 nanofiber.

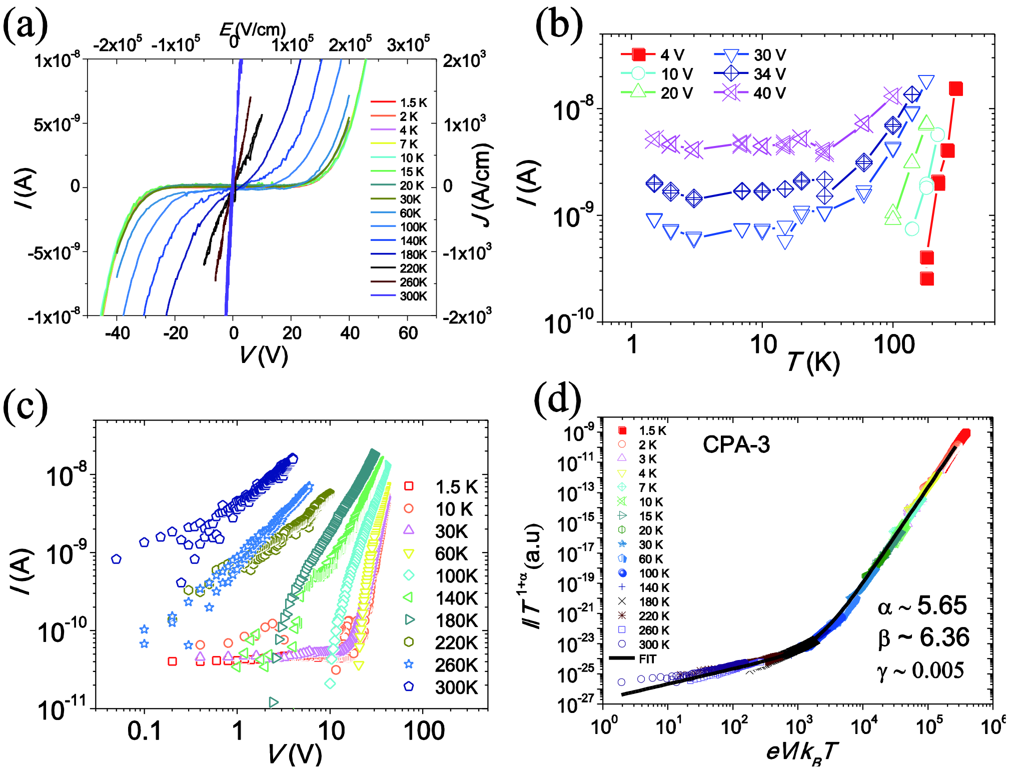

Figure. S7. (a) Temperature dependence of *I-V* characteristics for CPA-3 nanofiber in linear scale. (b) Temperature dependence of current at various source-drain applied voltages. As voltage increases temperature dependence weakens and deviates from power law dependence at low voltages. (c) Temperature dependence of *I-V* characteristics plotted in double logarithmic scale shows quasi power law behavior. (d) *I-V* curves from different temperatures collapsed in a single universal curve. α is the exponent of the temperature dependence of conductivity, $G(T) \propto T^{\alpha}$ and β is obtained from the fitting.

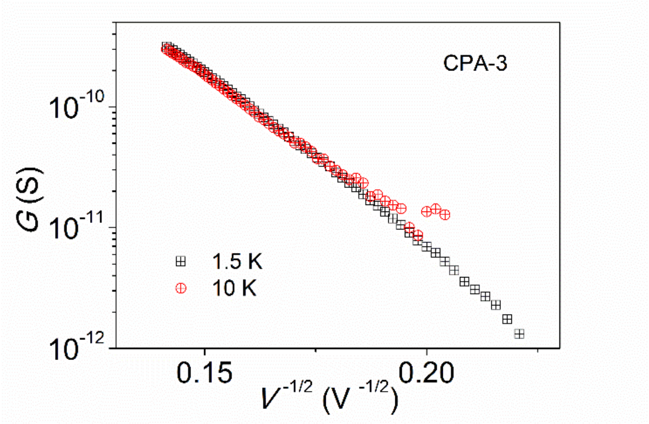

Figure. S8. The bias voltage dependence of the non-Ohmic conductance at low temperatures (T < 10 K) and high voltages (V > ~20 V) shows the characteristic of ES-VRH, $G(V) \propto \exp\left[-(T_0/V)^{1/2}\right]$ in the CPA-3 nanofiber.

3. Measurements of the CPANI-1 nanofiber

Figure S9 shows the AFM topography of the CPANI-1 nanofiber with Ti/Au (5/95 nm) top contact. The nanofibrila morphology is retained after pyrolysis as shown in Figure S9. Measurements are performed in the bottom two electrodes with two probe geometry. The distance between electrodes is 1 μm. Between the bottom two electrodes, the CPANI-1 nanofiber consists of 5 small parallel nanofibers which have diameters of 10 ~ 15 nm. Room temperature resistance and conductivity of the CPANI-1 nanofiber was 1 MΩ and 2 S/cm.

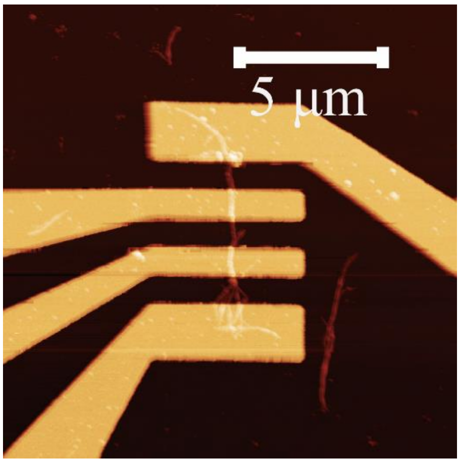

Figure. S9. AFM topography of CPANI-1 nanofiber with Ti/Au top contact (Measurements on bottom two electrodes)

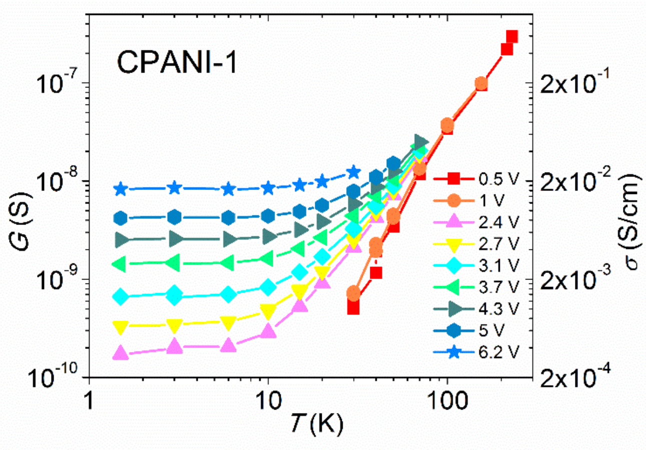

Figure. S10. Temperature dependence of conductance at various fixed voltages of the CPANI-1 nanofiber is presented in log-log scale. At 0.5 and 1 V, conductance strongly depends on temperature and follows power law. As voltage increases and temperature lowers, temperature dependence tends to decrease, which supports the hopping transport where the activation-less hopping occurs at low temperature and electric field compensates for activation energy at high voltages.

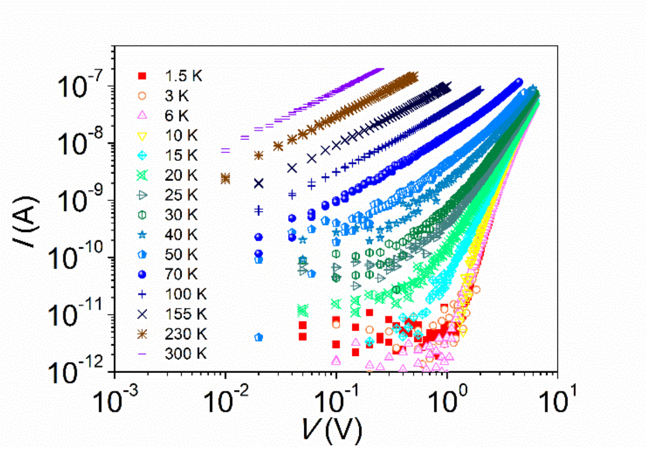

Figure. S11. *I-V* in log-log scale of the CPANI-1 nanofiber follows apparent power law behaviors. The power law at low temperature is apparent after threshold voltages but not strict as the slope tends to decrease at high voltages.

4. Measurements for cross-junction between CPA-2 and CPA-3 nanofibers

A cross-junction of the two different CPA nanofibers is formed accidentally during dispersion on the substrate as in Figure S4. Electrodes B-C is measured to characterize the cross-junction between the CPA-2 and the CPA-3 nanofibers. The cross-junction of the CPA-2 and the CPA-3 consists of the 510 nm length of the CPA-2 nanofiber with diameter 25 nm and the 870 nm length of the CPA-3 nanofiber with diameter 25 nm. Room temperature resistance of the junction between the two nanofibers was 210 MΩ.

Figure S(12a) shows temperature dependence of *I-V* characteristics for the cross-junction between the CPA-2 and the CPA-3 nanofibers (B-C in Figure S4) measured in 2 probe geometry. Interestingly, it shows similar behaviors as those of CPA nanofibers without a junction such as non-linearity, transport gap and weak temperature dependence at low temperatures. Other similar behaviors such as power law behavior of conductance at low bias and deviation from power law at high bias voltages [Figure S(12b)], quasi power law behavior of *I-V* [Figure S(12 c)] and the scaling behavior of *I-V* curves [Figure S(12d)] are also observed. It is interesting that the same scaling behavior is observed in a cross-junction of CPA nanofibers, which indicates that electrical transport through the cross-junction is not dominating the overall transport between the two electrodes. This finding also supports the hopping conduction in carbonized polymers, not the Luttinger liquid transport.

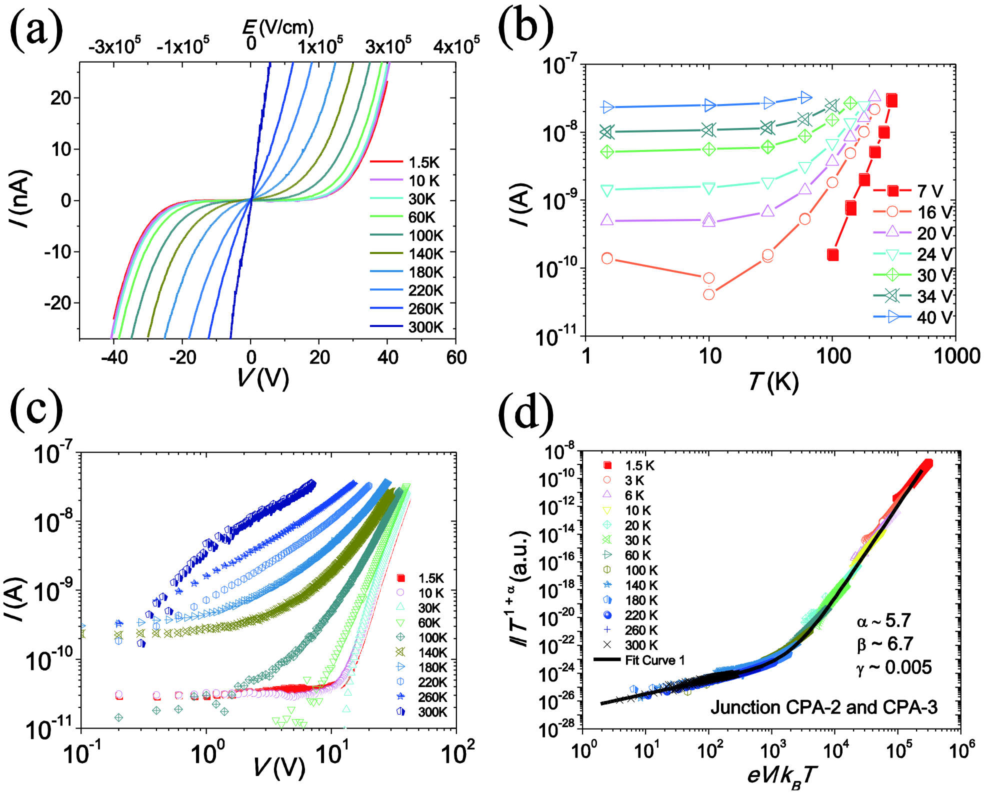

Figure. S12. (a) Temperature dependence of *I-V* characteristics in linear scale for the cross-junction between the CPA-2 and the CPA-3 nanofibers. (b) Temperature dependence of current at various source-drain applied voltages. As voltage increases temperature dependence weakens and deviates from power law dependence at low voltages. (c) Temperature dependence of *I-V* characteristics plotted in double logarithmic scale shows quasi power law behavior. (d) *I-V* curves from different temperatures collapsed in a single universal curve. α is the exponent of the temperature dependence of conductivity, $G(T) \propto T^{\alpha}$ and β is obtained from the fitting.

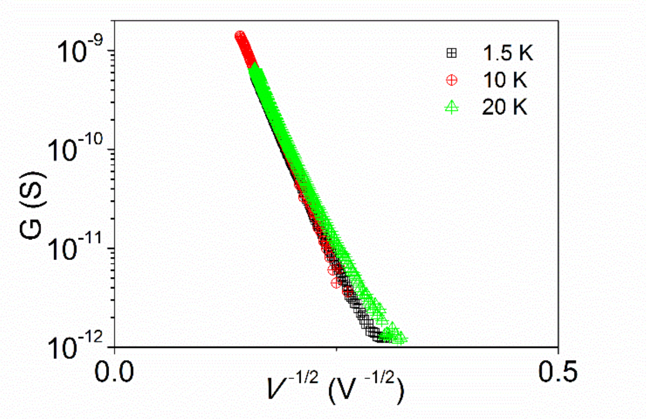

Figure. S13. The bias voltage dependence of the non-Ohmic conductance at low temperatures (T < 20 K) and high voltages (V > ~10 V) shows the characteristic of ES-VRH, $G(V) \propto \exp\left[-(T_0/V)^{1/2}\right]$ in the cross-junction between the CPA-2 and the CPA-3 nanofibers.

5. Applicability of Coulomb blockade model for non-conducting region at low temperatures

(1) Evaluation of threshold voltages

Threshold voltages are evaluated by plotting I*dV/dI vs V. Figure S14 and Figure S15 shows I*dV/dI vs V plot of CPA and CPANI nanofibers[1].

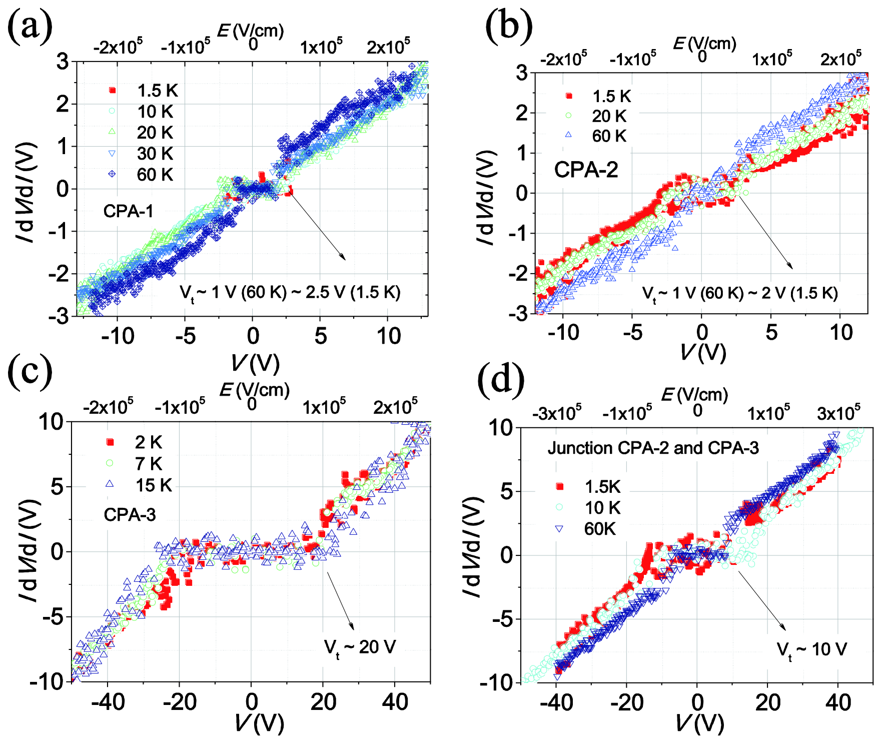

Figure. S14. I*dV/dI vs V plot of the CPA-1 (a), CPA-2 (b), CPA-3 (c), and the cross-junction between the CPA-2 and the CPA-3 nanofibers (d) and evaluated threshold voltages.

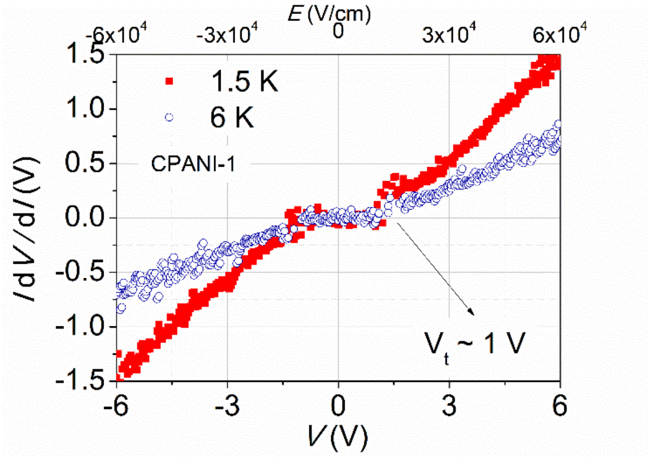

Figure. S15. I*dV/dI vs V plot of the CPANI-1 nanofiber at 1.5 K and 6 K. Threshold voltage is around 1 V.

(2) Relevance of Coulomb blockade model

Figure S16 shows current vs $(V-V_t)/V_t$ plot in double logarithmic scale for (a) CPA-1, (b) CPA-2, (c) CPA-3, and (d) CPANI-1 nanofiber. ζ values in $I\sim[(V-V_t)/V_t]^{\zeta}$ were obtained from slopes and shown in Figure S16. ζ is predicted to be with $\zeta\sim1$ in 1-D and $\zeta\sim2$ or 5/3 in 2-D array of small quantum dots[1,2]. In our nanofibers, slopes vary from 0.6 to 4.0 as shown in Figure S16. At low voltages, ζ varies smoothly so that it is hard to define ζ. For the CPA-1 nanofiber, ζ varies from 0.6 to 1.1 at low voltages but for other samples ζ becomes zero at low voltages and gradually increases. At high voltages ζ varies from 3.7 to 4.1 for the CPA-1 nanofiber and 3.9 to 4.0 for the CPA-2 nanofiber. ζ was higher than 2 both in the CPA-3 and the CPANI-1 nanofibers. However, higher values of exponents than theory is usual and cannot rule out the Coulomb blockade. The charging energy argument in the main text shows that the threshold voltage should exist even at room temperature, which contradicts to our data. The sp2 aromatic rings exist as confirmed by appearance of G and D bands [Fig. (1b)]. But the broadness of Raman bands suggests that the aromatic rings do not form well-defined crystalline islands yet but randomly distributed in the sample. This point is different from other reported Coulomb blockade systems[3,4] where well-defined crystalline islands exist for

Coulomb blockade transport.

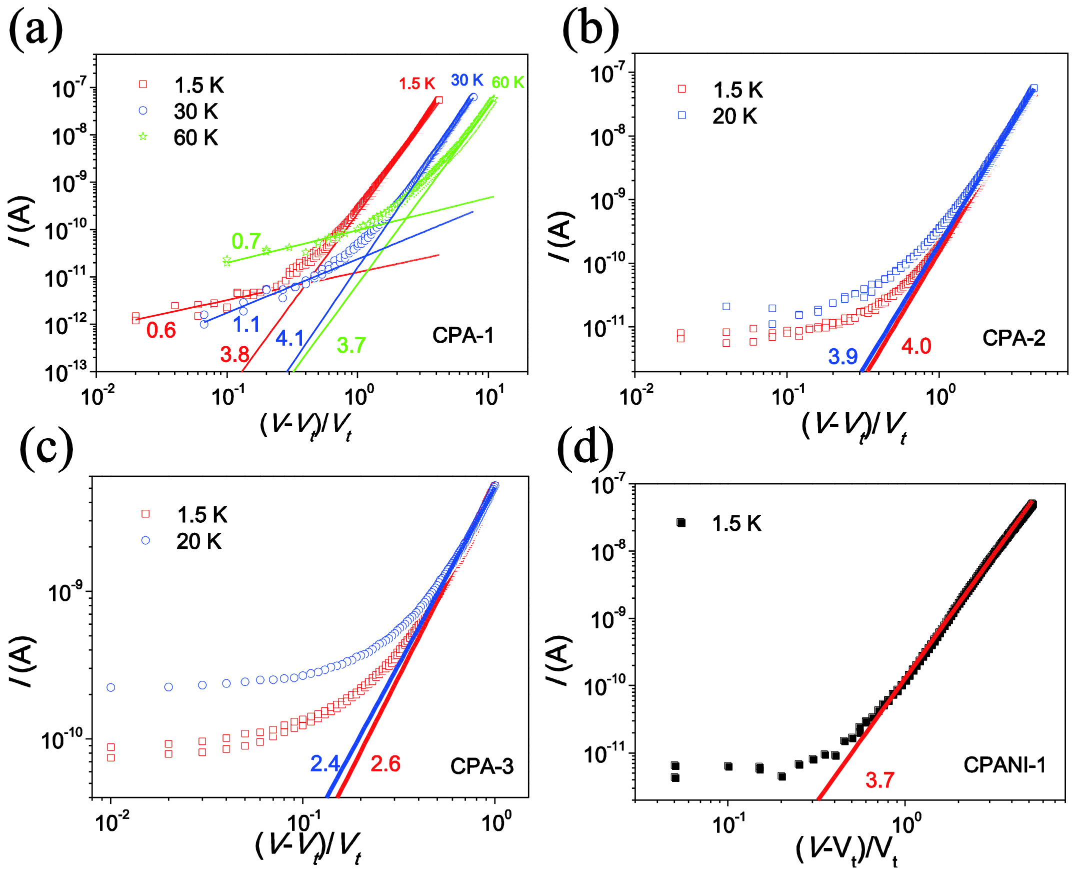

Figure. S16. Current vs $(V\text{-}V_t)/V_t$ plot in double logarithmic scale for (a) CPA-1, (b) CPA-2, (c) CPA-3, and (d) CPANI-1 nanofiber. Slopes determining ζ in $I \propto [(V - V_t)/V_t]^{\zeta}$ are shown in each figure.

7. Contact resistance

Poor interfaces or work function mismatch might lead to high contact resistances and Schottky barriers which display themselves temperature-dependent non-linear *I-V* characteristic. At low temperatures, 4 probe measurements are deliberately avoided due to high resistances of carbonized polymer nanofibers. Meanwhile, at room temperature, we have measured and compared 2 probe (R_{2P}) and 4 probe (R_{4P}) resistances using different metal contacts (Ti/Au, Ni, Pd). The contact resistance obtained as $R_c = R_{2p} - R_{4p}$ is usually small relative to the high sample resistance regardless of the metal used for contact. Therefore,

contributions from the contact is not significant compared to the sample resistance. This relatively small contact resistance is presumably due to the fact that we contact wide metal electrodes with 2 μm width on top of fibers using evaporation and the sample resistance is significantly high enough. Also the symmetric *I-V* at all temperatures (Figure 1c and 1d) is hardly originated from Schottky barriers. If the tunneling through Schottky barriers is dominant, the *I-V* is asymmetric as the two Schottky barriers at the source and drain electrodes cannot be the same. Therefore, we conclude that the non-linear *I-V* is not originated from Schottky barrier.

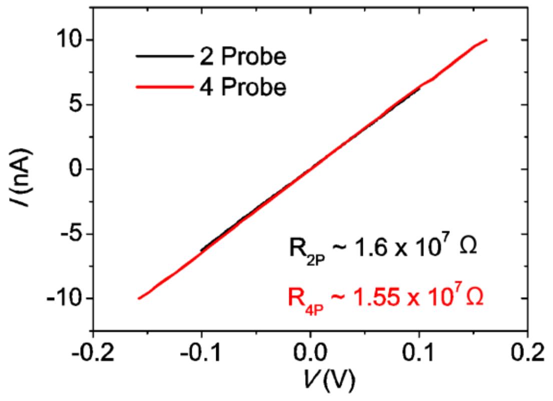

Figure. S17. 2 probe and 4 probe measurements of a typical carbonized polymer nanofiber at room temperature. The contact resistance is small compared to the high sample resistance**.**

References

[1] Aleshin, A. N., Lee, H. J., Jhang, S. H., Kim, H. S., Akagi, K., and Park, Y. W. Coulomb-blockade transport in quasi-one-dimensional polymer nanofibers. Phys. Rev. B **72**, 153202 (2005).

[2] Middleton, A. A. & Wingreen, N. S. Collective transport in arrays of small metallic dots. Phys. Rev. Lett. **71**, 3198 (1993).

[3] Parthasarathy, R., Lin, X.-M. & Jaeger, H. M. Electronic transport in metal nanocrystal arrays: The effect of structural disorder on scaling behavior. Phys. Rev. Lett. **87**, 186807 (2001).

[4] Akai-Kasaya, M., Okuaki, Y., Nagano, S., Mitani, T. & Kuwahara, Y. Coulomb blockade in a two-dimensional conductive polymer monolayer. Phys. Rev. Lett. **115**, 196801 (2015).
